# Prevalence of papillary muscle hypertrophy in fabry disease

**DOI:** 10.1186/s12872-023-03463-w

**Published:** 2023-08-27

**Authors:** Tomás Francisco Cianciulli, María Cristina Saccheri, Mariano Napoli Llobera, Lorena Romina Balletti, Matín Alejandro Beck, Luis Alberto Morita, Jorge Alberto Lax

**Affiliations:** 1 Division of Cardiology, Echocardiography Laboratory, Hospital of the Government of the City of Buenos Aires “Dr. Cosme Argerich”, Ciudad Autónoma de Buenos Aires, Argentina; 2 Researcher of the Ministry of Health, Government of the City of Buenos Aires, Ciudad Autónoma de Buenos Aires, Argentina

**Keywords:** Fabry disease, Left ventricular hypertrophy, Papillary muscle hypertrophy

## Abstract

**Background and aims:**

Fabry disease (FD) is an X-linked genetic lysosomal disease, in which a deficit in the alpha-galactosidase A enzyme results in lysosomal build-up of globotriaosylceramide in several organs, causing cardiac, renal and cerebrovascular complications. The aim of this study was to assess the prevalence of papillary muscle hypertrophy (PMH) in patients with FD.

**Methods:**

A group of 63 patients with FD and a positive genetic diagnosis were studied and were divided into two groups: one included 24 patients with FD and LVH and another group included 39 patients with FD and without LVH. Papillary muscles were measured from the left parasternal short axis view, defining PMH as a diastolic thickness greater than 11 mm in any diameter.

**Results:**

Patients with FD and LVH had a high prevalence of anterolateral PMH (66.6%), and such prevalence was lower for the posteromedial PMH (33.3%). However, patients who had not yet developed LVH had a high prevalence of anterolateral PMH (33.3%).

**Conclusions:**

Patients with FD in the pre-clinical stage (without LVH) have a high prevalence of PMH, especially involving the anterolateral papillary muscle. This finding could be an early marker for the development of LVH, allowing to suspect the disease during its early stages, and begin enzyme replacement therapy in the appropriate patients.

## Introduction

Fabry disease (FD) is caused by a recessive, X-linked genetic defect, which leads to a deficit in the activity of a lysosomal enzyme, α-galactosidase A [1]. As a result of this deficiency, glycosphingolipids, mainly globotriaosylceramide (Gb3), accumulate in lysosomes of various organs and systems. Due to this massive tissue build-up, many patients die between the fourth and fifth decades of life due to renal, cardiac or cerebrovascular complications. Currently, the most frequent cause of death in patients with FD is of cardiac origin, due to heart failure or sudden death [2–4].

Regarding its prevalence, FD is the 2nd most frequent storage disorder (1 case per 40.000 males), after Gaucher’s disease. Cardiac involvement occurs frequently in FD, and occasionally the heart is the only affected organ. The characteristic phenotype is symmetric left ventricular hypertrophy (LVH), that may mimic hypertrophic symmetric non-obstructive cardiomyopathy (HCM) [5, 6].The presence of a dynamic subaortic gradient supports the diagnosis of hypertrophic cardiomyopathy and would allow to rule out FD.

According to the European Registry of Cardiomyopathies [7] and the Argentine Consensus on CMP [8], amyloidosis is the most frequent phenocopy, followed by FD, hence, the differential diagnosis is very important in order to prescribe a specific treatment. Interest about the diagnosis of FD has grown since 2002, when enzyme replacement therapy (ERT) became available. When ERT is administered early, partial remission of the disease may occur and long-term complications are prevented, especially reducing or delaying the progression of LVH [9].

Since the clinical presentation is variable, it is difficult to make an early diagnosis and the mean diagnostic delay is 15 to 20 years after the beginning of symptoms.

In patients with FD, a targeted history taking, physical exam, laboratory workup to assess enzyme levels, and genetic testing are the main pillars for the differential diagnosis of the disease.

Symptoms of FD reflect the various organs affected (acroparesthesias, angiokeratomas, hypohydrosis, proteinuria that may progress to chronic renal failure, gastrointestinal symptoms, transient ischemic accident or stroke). However, in spite of early multisystemic manifestations, the diagnosis is currently made late and many cases are still diagnosed as non-obstructive HCM.

Hence, a high level of suspicion is required for the diagnosis of FD, especially in patients with a normal 2-D echocardiogram.

The goal of this study was to demonstrate that FD can be suspected in patients with papillary muscle hypertrophy and an otherwise normal 2-D echocardiogram.

That would allow to detect early cardiac involvement in patients with FD without LVH, and decide whether they require enzyme replacement therapy to prevent irreversible damage to the heart, kidney and brain.

## Objective

To assess the prevalence of papillary muscle hypertrophy in FD.

## Materials and methods

This was a single-center, cross-sectional study, which assessed the presence of papillary muscle hypertrophy (PMH) in patients with FD, with and without LVH.

Thirty-nine patients with a diagnosis of FD without LVH and 24 patients with FD and LVH were included.

### Inclusion criteria

Patients included had an enzymatic and genetic diagnosis of FD, and were aged 18 years or older, with or without LVH. Their α-galactosidase A levels had to be reduced or absent, and a chromosome X-linked mutation was present (locus Xq 22.1).

The normal value of α-galactosidase A enzymatic activity is greater than 5 µmol/l/hour. An α-galactosidase A level below 1 µmol /l/hour was required for the diagnosis of FD.

The normal value of α-galactosidase A in peripheral blood leukocytes, measured with the fluorometric method, is 395 to 780 nmol/mg/hour. A value below 80 nmol/mg/h was required for the diagnosis of FD.

### Exclusion criteria

Exclusion criteria were a history of coronary, valvular or pericardial disease, congenital heart disease, hypertension, diabetes mellitus, anemia, asthma, chronic obstructive pulmonary disease, thyroid dysfunction, renal failure, pregnancy, alcoholism, patients younger than 18 years old, and patients with a poor ultrasound window.

The study was approved by the Research and Ethics Committee of the Hospital “Dr. Cosme Argerich”. Written consent for submission and publication of this study, including images and associated text, has been obtained from the patient in line with COPE guidance.

### 2-D Echocardiography

The 2-D echocardiogram was performed with a Vivid 7 machine (General Electric Medical Systems) and a 1.5 to 4 MHz transducer.

Left parasternal (long and short axes) as well as apical 2-, 3- and 4-chamber views were obtained. Two-D and M-mode images were obtained from the left parasternal short axis view at the papillary muscle level, and the following measurements were made according to the American and European Societies of Echocardiography: end-diastolic LV diameter (LVEDD), end-systolic LV diameter (LVESD), diastolic thicknesses of the ventricular septum and LV posterior wall, right ventricular and left atrial (LA) diameters [10, 11].

**Fig. 1:**
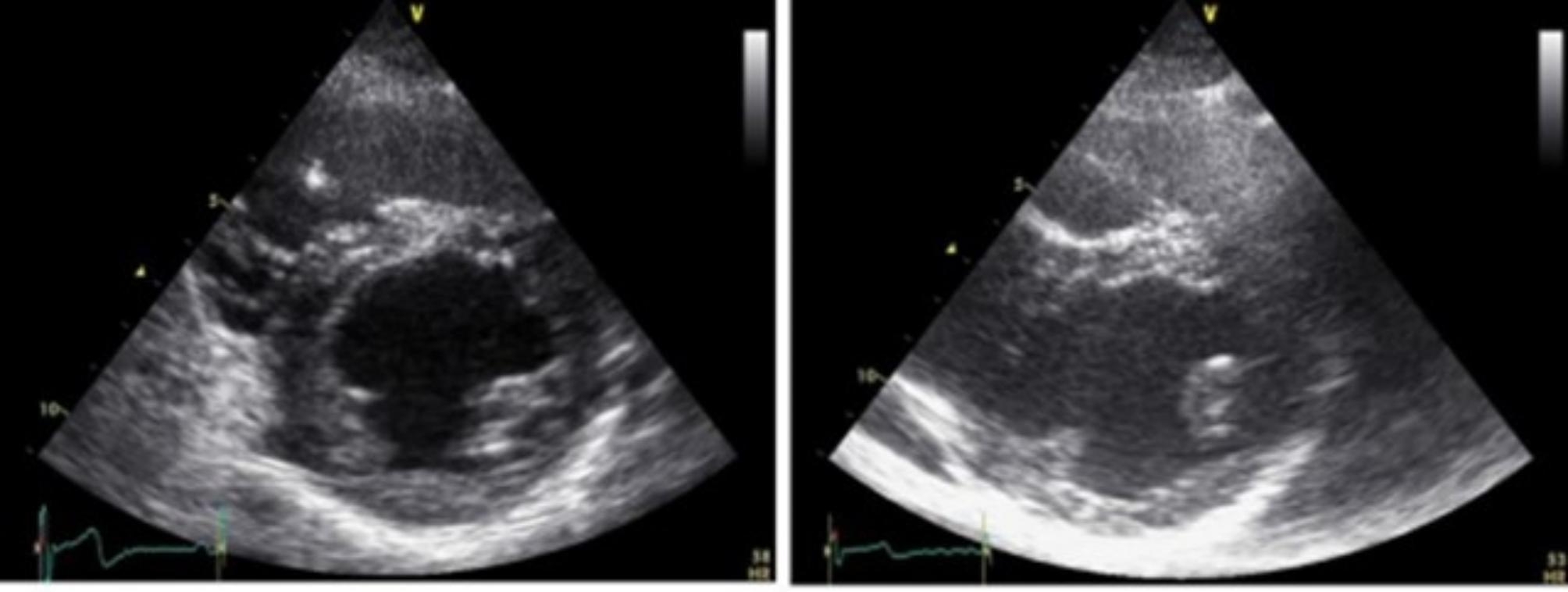
Patient with Fabry disease without left ventricular hypertrophy. Two-dimensional Echocardiogram. Left parasternal short axis at end-diastole. Left panel: shows normal posteromedial and anterolateral papillary muscles. Right panel: shows hypertrophy of the anterolateral papillary muscle, without left ventricular hypertrophy

**Fig. 2:**
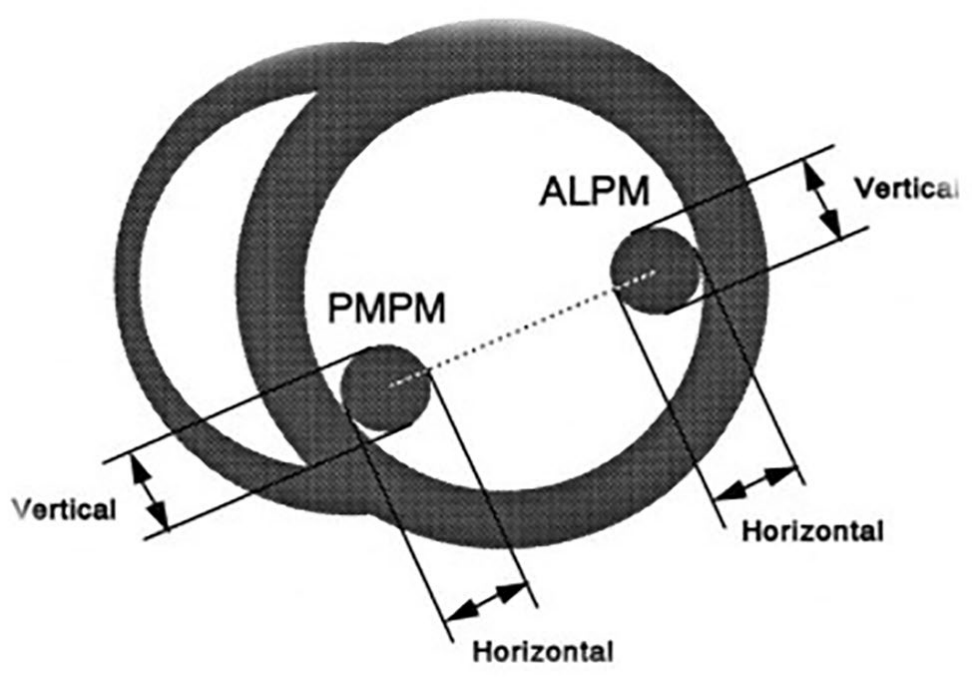
Diagram showing measurements of the horizontal and vertical diameters of both papillary muscles, obtained from the left parasternal short axis at end-diastole. PMPM = posteromedial papillary muscle; ALPM = anterolateral papillary muscle. Left ventricular shortening fraction (SF) was estimated using the following formula: [(LVDD − LVSD) / LVDD] × 100. The LV mass index (LVMI) was calculated using the Devereux formula: 0.8 *([LVDD + VSD + PWD)^3^ – LVDDI 3] x 1.04) + 0.6/ Body surface

Both diameters of each papillary muscle were measured at the end diastole in the left parasternal short axis view. Hypertrophy of the papillary muscles was diagnosed when the horizontal or vertical diameters measured more than 11 mm (Figs. 1 and 2) [12].

### Reproducibility

To assess interobserver variability, two independent investigators performed a 2-D echocardiogram with the appropriate measurements in 10 patients included in the study, in a period < 24 h to avoid differences attributable to variations in hemodynamic status.

Subsequently, after a 24-hour interval, one of the investigators re-analyzed the echocardiographic parameters in the images obtained, to assess the intraobserver variability.

### Statistical analysis

Quantitative data with a normal distribution were expressed as means ± standard deviation and data with non-gaussian distribution were expressed as medians and inter-quartile intervals. The Student *t* test for paired data was used to compare quantitative variables with a normal distribution, and for variables with a non-normal distribution we used the Wilcoxon Signed Rank Test. Statistical analyses were performed with Statistix 7.0. software and a p value < 0.05 was considered significant.

## Results

We included 66 patients with an enzymatic and genetic diagnosis of FD. Among them, 3 patients were excluded due to a poor ultrasound window.

The final study population comprised a total of 63 patients, 24 patients with FD and LVH, and 39 patients with FD without LVH. As shown in Table 1, the group with FD and LVH was older and had a higher prevalence of male sex (p < 0.05).

Regarding conventional echocardiographic measurements, the group with LVH had greater wall thickness, LV mass and LA size (p < 0.05). Although LVDD was normal in both groups, patients with LVH had a larger diameter (p < 0.05). No significant difference was seen in RV dimension, SF, or LVEF.

**Fig. 3:**
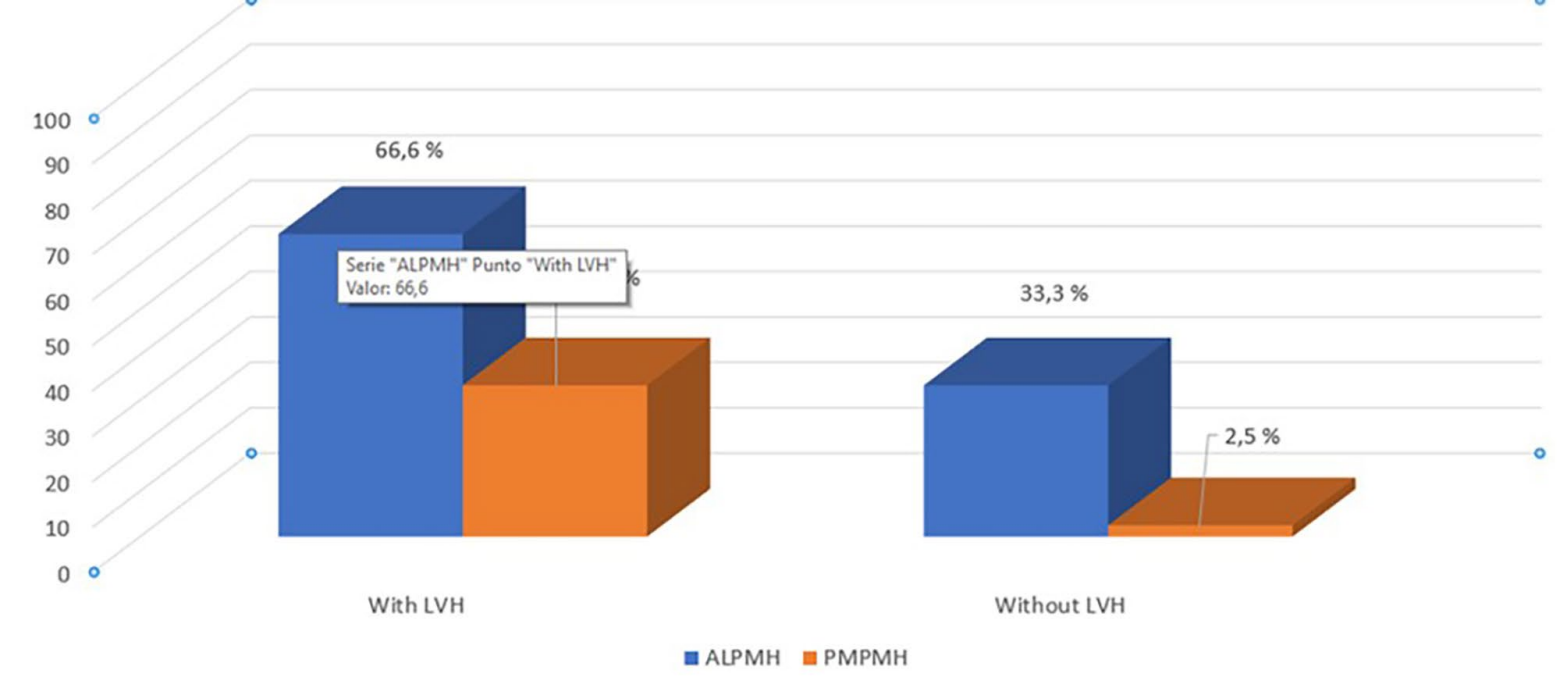
Prevalence of Papillary Muscle Hypertrophy in Fabry Disease. PMH = Papillary Muscle Hypertrophy, LVH = Left Ventricular Hypertrophy, ALPMH = Anterolateral Papillary Muscle Hypertrophy, PMPMH = Posteromedial Papillary Muscle Hypertrophy

Patients with FD and LVH had a higher prevalence of anterolateral PMH (66.6%; p = 0.02) and to a lesser extent posteromedial PMH (33.3%; p = 0.001). However, the group of patients who had not yet developed LVH exhibited a high prevalence of anterolateral PMH (33.3%). Table 1; Fig. 3.

**Table 1:** Baseline Demographic Characteristics and Echocardiographic Parameters in Patients with FD, with and without LVH

	FD with LVH (n = 24)	FD without LVH (n = 39)	p
Age (years)	45 ± 15	25 ± 9	< 0.05
Male Sex (%)	14 (58%)	9 (23%)	< 0.05
Right Ventricle (mm)	16.3 ± 6	18.5 ± 3	NS
LVDD (mm)	52.1 ± 8	45.8 ± 5	< 0.05
SF (%)	41 ± 15	45 ± 8	NS
EF (%)	69 ± 19	73 ± 7	NS
Ventricular Septum (mm)	19 ± 3	8.5 ± 0.8	< 0.05
Posterior wall (mm)	14 ± 2	6.2 ± 0.8	< 0.05
LVMI (g/m^2^)	293 ± 61	75 ± 12	< 0.05
LA (mm)	44.9 ± 6	31.5 ± 5	< 0.05
Anterolateral Papillary Muscle Hypertrophy (%)	16 (66.6)	13 (33.3)	0.02
Posteromedial Papillary Muscle Hypertrophy (%)	8 (33.3)	1 (2.56)	0.001

**Note**: Values are expressed as number and % of patients, or as mean ± standard deviation. FD = Fabry disease; LVH = left ventricular hypertrophy; NS = non-significant; LVDD = left ventricular diastolic diameter; SF = shortening fraction; EF = ejection fraction; LVMI = left ventricular mass index; LA = left atrium

## Discussion

In FD the catabolism of glycosphingolipids (Gb3) is altered, due to a deficit of the alpha-galactosidase A enzyme, resulting in systemic buildup of lysosomal Gb3 [13].

Phenotypes of FD vary, from the “classic” form which begins early during childhood and affects multiple organs, to the late form, where the heart is predominantly affected [14].

Accumulation of Gb3 in cardiomyocytes, the conduction system, vascular endothelium and fibroblasts leads to cellular and vascular dysfunction, causing cardiac manifestations. These include ventricular hypertrophy, progressive diastolic and systolic LV dysfunction, bradyarrhythmias and tachyarrhythmias, microvascular ischemia and mild valve dysfunction [14, 15].

Skeletal muscle abnormality is frequently documented among individuals diagnosed with FD. Muscular symptoms stand out as a primary manifestation of FD, emerging before the onset of both cardiomyopathy and chronic kidney dysfunction. It remains uncertain whether this stems from the same cardiac dysfunction or from direct muscular involvement. In younger patients, muscle myocytes are usually unaffected, while blood vessels show the presence of mild glycosphingolipid accumulation in endothelial and smooth muscle cells. On the other hand, in older patients, signs of myopathy are detected in electromyography, along with an increase in left ventricular mass [16].

Recently, a high glycolytic rate and underutilization of lipids as an energy fuel have been observed in FD patients. In the pursuit of a potential mechanism, an upregulation of HIF-1 (hypoxia-inducible factor-1) has been identified in both mice and FD patients, leading to a reversal of cellular metabolic remodeling, revealing a Warburg effect. Blood lactate has surfaced as a potent biosensor of this metabolic disruption, introducing a novel prospect as a diagnostic and monitoring tool [17].

Left ventricular hypertrophy is a maladaptive and non-specific cardiac response to a great variety of stimuli in many cardiac and systemic disorders [18] It is a key finding in FD and is reported in up to 50% of affected men and one third of women [19, 20].

The mild to moderate impairment in diastolic filling is a relatively common finding, and probably represents the most important cause of dyspnea in patients with FD. Valve structural abnormalities are frequent due to valve infiltration [21].

The LVH is usually concentric, without LV outflow tract obstruction. However, in severe cases an asymmetric variant may be found, with septal thickening and fibrotic thinning of the posterior wall, characteristics that are unique to FD [21] Right ventricular (RV) hypertrophy is also common and may progress to ventricular dilation [14].

Several review papers and case reports on FD have shown that patients with Fabry cardiomyopathy have a high prevalence of PMH [18, 21, 22]. This finding could hence be useful to distinguish cardiomyopathy due to FD from hypertrophic cardiomyopathy. Our study confirms that in advanced stages of FD (Fabry cardiomyopathy) there is high prevalence of PMH. Additionally, our study detected that in patients with FD without LVH there is also high prevalence of anterolateral PMH, therefore that finding could be used as an early marker for the diagnosis of FD.

Other cardiac diseases that cause LVH also result in PMH. For example, in hypertensive cardiac disease, stress leads to an increase in wall thickness, which may be accompanied by an increased size of the PM during the last stage of the disease, albeit not as marked as in FD. Friedreich’s ataxia is a mitochondrial disorder that causes LVH. However, wall thickness rarely exceeds 14 mm and therefore, PM size does not increase as much. In cardiac amyloidosis LVH is also relatively frequent, although the PM do no exhibit similar degrees of hypertrophy [9].

Only in FD cardiomyopathy LVH and PMH are seen associated with a relatively small LV cavity, resulting in an absolute and relative increase in PM size. 9].

Two-D strain allows detection of early abnormalities in FD without myocardial hypertrophy, when the longitudinal strain of the LV is ≥-15% in at least one segment. Possibly in FD, beyond the lysosomal storage of glycosphingolipids or the abnormal synthesis of sarcomeric proteins, there is interference in sarcomeric contraction and relaxation, resulting in a decrease in longitudinal myocardial strain [22, 23]. Left atrial strain is also decreased in FD, hence, adding left atrial strain to an echocardiogram with LVH of unclear etiology may be useful to identify FD as a potential cause [24].

Currently, cardiac magnetic resonance (CMR) with tissue characterization and T1 and T2 mapping techniques, contributes to the diagnosis and suspicion of FD [25]. Also, the level of image resolution required for an accurate measurement of LV mass and PM can only be achieved with CMR [26].

The accumulation of sphingolipids at the myocardial level can be detected using native T1 mapping. Typically, the low native T1 value precedes the development of the classic sign of LVH, making it a considered early marker of the disease. Myocardial native T1 captures signals from both myocytes (with sphingolipids) and intramyocardial blood. Blood has a longer and more variable T1 value, and the contaminating signal acts as noise, diminishing the precision and accuracy of myocardial T1 measurement for sphingolipid accumulation in myocytes. However, blood’s T1 can be independently measured in the cardiac blood pool, allowing for a correction strategy. This blood-based correction of native myocardial T1 has been observed to increase the proportion of subjects with FD who exhibit a low myocardial T1 value [27].

However, echocardiography is a simple and widely available method for the assessment of ventricular geometry and specific myocardial structures, used as diagnostic markers for several cardiac diseases [9].

Nowadays, echocardiography and CMR are complementary examinations that aid in the diagnosis and monitoring of Fabry disease treatment. This includes identifying the specific cardiac phenotype of FD, distinguishing it from other types of left ventricular hypertrophy (LVH), and selecting suitable patients for therapeutic interventions. Advances in cardiac imaging hold the promise of identifying Fabry disease-related abnormalities in their early stages, thereby facilitating early prognostic characterization [28].

The presence of PMH in patients without LVH could allow to suspect the disease in its early stages, and early treatment could allow for partial remission of the disease and prevention of its long-term complications.

### Limitations

Although 2-D echocardiography is the standard diagnostic tool in daily cardiology practice, papillary muscles are three-dimensional structures that may not be completely evaluated with a two-dimensional method, and that is a limitation of the method.

Since the study was conducted at a single center, the results might not be representative of the genetic and ethnic diversity of other populations.

We acknowledge that the number of patients in our study is small, which might limit the generalizability of the results to a larger population. FD is an infrequent disorder, with an incidence of 1/40.000 men. Thus, our study population represents 11.6% of the estimated number of patients with FD in our country. A larger sample size could provide a more comprehensive picture of the prevalence of PMH in FD patients.

The study design is cross-sectional, preventing the establishment of causal relationships between the presence of PMH and the disease’s progression over time. A longitudinal study could offer deeper insights into this association.

Included patients had a positive genetic diagnosis of FD. This might introduce selection bias, as patients with more severe forms of the disease could be more likely to undergo genetic testing and thus be overrepresented in the sample.

## Conclusions

Patients with FD in the preclinical stage (without LVH) have a high prevalence of PMH, especially the anterolateral PM, and that could be a predictor for the development of LVH.

These findings could provide useful information regarding the pathophysiology of cardiac involvement in FD and help understand whether early enzyme replacement therapy might be useful in modifying the clinical course and prognosis of patients with this disease.

## Data Availability

The datasets used and analyzed during the current study available from the corresponding author on reasonable request.

## References

[1] Mehta A, Ricci R, Widmer U, Dehout F (2004). Fabry disease defined: baseline clinical manifestations of 366 patients in the Fabry Outcome Survey. Eur J Clin Invest.

[2] MacDermot KD, Holmes A, Miners AH (2001). Anderson-Fabry disease: clinical manifestations and impact of disease in a cohort of 60 obligate carrier females. J Med Genet.

[3] MacDermot KD, Holmes A, Miners AH (2001). Anderson-Fabry disease: clinical manifestations and impact of disease in a cohort of 98 hemizygous males. J Med Genet.

[4] Meikle PJ, Hopwood JJ, Clague AE (1999). Prevalence of lysosomal storage disorders. JAMA.

[5] Desnick RJ, Loannou YA, Eng CM (2001). a-Galactosidase A deficiency: fabry disease. The metabolic and molecular bases of inherited disease.

[6] Brady RO, Gal AE, Bradley RM, Martensson E (1967). Enzymatic defect in Fabry’s disease. Ceramidetrihexosidase deficiency. N Engl J Med.

[7] Elliott P, Charron P, Blanes JR, Tavazzi L (2016). EORP Cardiomyopathy Registry Pilot Investigators. European Cardiomyopathy Pilot Registry: EURObservational Research Programme of the European Society of Cardiology. Eur Heart J.

[8] Saccheri MC, Cianciulli TF (2016). Consenso Argentino de Miocardiopatías Hipertróficas. Rev Argent Cardiol.

[9] Nieman M, Liu D, Hu K, Herrmann S (2011). Prominent papillary muscles in fabry disease: a diagnostic marker?. Ultrasound in Med & Biol.

[10] Leitman M, Lysyansky P, Sidenko S, Shir V (2004). Two-dimensional strain-a novel software for real-time quantitative echocardiographic assessment of myocardial function. J Am Soc Echocardiogr.

[11] Lang RM, Badano LP, Mor-Avi V, Afilalo J (2015). Recommendations for Cardiac Chamber quantification by Echocardiography in adults: an update from the American Society of Echocardiography and the European Association of Cardiovascular Imaging. J Am Soc Echocardiogr.

[12] Kobashi A, Suwa M, Ito T, Otake Y (1998). Solitary papillary muscle hypertrophy as a possible form of hypertrophic cardiomyopathy. Jpn Circ J.

[13] Yousef Z, Elliott PM, Cecchi F, Escoubet B (2013). Left ventricular hypertrophy in fabry disease: a practical approach to diagnosis. Eur Heart J.

[14] Namdar M (2016). Electrocardiographic changes and arrhythmia in fabry disease. Front Cardiovasc Med.

[15] Patel V, O’Mahony C, Hughes D (2015). Clinical and genetic predictors of major cardiac events in patients with Anderson-Fabry disease. Heart.

[16] Chimenti C, Padua L, Frustaci A (2012). Cardiac and skeletal myopathy in fabry disease: a clinicopathologic correlative study. Hum Pathol.

[17] Gambardella J, Fiordelisi A, Cerasuolo FA, Buonaiuto A (2023). Experimental evidence and clinical implications of Warburg effect in the skeletal muscle of fabry disease. iScience.

[18] Widemann F, Störk S, Herrmann S, Ertl G (2011). The various forms of left ventricular hypertrophy: diagnostic value of echocardiography. Herz.

[19] Kampmann C, Linhart A, Baehner F, Palecek T (2008). Onset and progression of the Anderson-Fabry disease related cardiomyopathy. Int J Cardiol.

[20] Linthorst GE, Bouwman MG, Wijburg FA, Aerts JM (2010). Screening for fabry disease in high-risk populations: a systematic review. J Med Genet.

[21] Linhart A, Lubanda JC, Palecek T, Bultas J (2001). Cardiac manifestations in fabry disease. J Inherit Metab Dis.

[22] Weidemann F, Wanner C, Breunig F (2008). Nome nest omen. Eur J Echocardiography.

[23] Saccheri MC, Cianciulli TF, Lax JA, Gagliardi JA (2013). Two-Dimensional Speckle Tracking Echocardiography for early detection of myocardial damage in Young Patients with Fabry Disease. Echocardiography.

[24] Frumkin D, Mattig I, Laule N, Al Daas M (2021). Comparative analysis of phasic left atrial strain and left ventricular posterolateral strain pattern to discriminate fabry cardiomyopathy from other forms of left ventricular hypertrophy. Echocardiography.

[25] Kozor R, Callaghan F, Tchan M, Hamilton-Craig C (2015). A disproportionate contribution of papillary muscles and trabeculations to total left ventricular mass makes choice of cardiovascular magnetic resonance analysis technique critical in fabry disease. J Cardiovasc Magn Reson.

[26] Kozor R, Nordin S, Treibel TA, Rosmini S (2017). Insight into hypertrophied hearts: a cardiovascular magnetic resonance study of papillary muscle mass and T1 mapping. Eur Heart J.

[27] Nickander J, Cole B, Nordin S, Vijapurapu R (2023). Increased cardiac involvement in fabry disease using blood-corrected native T1 mapping. Sci Rep.

[28] Perry R, Shah R, Saiedi M, Patil S (2019). The role of Cardiac Imaging in the diagnosis and management of Anderson-Fabry Disease. J Am Coll Cardiol Img.

